# COMMENTARY ON: COMPARISON OF MOTION SENSOR AND HEART RATE MONITOR FOR ASSESSMENT OF PHYSICAL ACTIVITY INTENSITY IN STROKE OUTPATIENT REHABILITATION SESSIONS: AN OBSERVATIONAL STUDY

**DOI:** 10.2340/jrm.v56.41967

**Published:** 2024-10-09

**Authors:** Felicianus Anthony PEREIRA, Asfa WAHEED, Javeria RAO, Muhammad UMAIR

**Affiliations:** Dow Institute of Physical Medicine & Rehabilitation, Dow University of Health Sciences, Karachi, Pakistan

We read with great interest the study by Goncalves et al. (1), which assessed the intensity of activity levels among stroke patients. We commend the authors for highlighting the importance of rehabilitation protocols in a population who face long-term complications of their disorder. With the long-term effects of stroke, neuroplasticity is of importance. Longer time to rehabilitation can negatively impact the recovery of people affected and can thus affect their ability to function independently, which can potentially reduce cardiovascular response. The study utilized various measurement parameters to assess levels of exercise intensity. Motion sensors and heart rate monitors were used to determine the intensity of physical activity among patients. After carefully reading through this study, we would like to offer a few points of discussion.

There appears to be a lack of homogeneity between participants. The severity of stroke is an important factor in the outcome of rehabilitation; however, it was not determined before enrolling participants in the study (only chronicity of stroke was mentioned). Further, the artery involved can lead to a difference in symptoms displayed by patients, which can also cause difficulties in rehabilitation. Cardiac complications are likely after the occurrence of stroke and the severity of cardiac consequences is directly proportional to the severity of ischaemic stroke and its consequences. Standardizing the participants involved would have yielded greater credibility to the study methodology and a better understanding of the results (2). A screening tool such as the NIHSS scale would have helped screen patients and assign them to groups for better results (3).

Another point of interest is the exercise protocols used for the participants. No standard exercise guideline was implemented in this study. Exercise guidelines for stroke recommend 3 to 5 days per week of aerobic training (20–40 min at moderate intensity) and 2 to 3 days per week of resistance training (1–3 sets of 8–15 repetitions between 30% and 50% of 1 repetition maximum) (4). Studies have shown that both aerobic and strength training exercises demonstrate a good prognosis in post-stroke patients (5). The authors focused on balance, endurance, and functional lower limb training in their study. Exercise protocols were also performed only twice, which may not provide sufficient time to determine the effects of exercise interventions, or patient adaptation to exercise (6). As the inclusion criteria included patients with chronic stroke, there are a variety of complications that could arise such as spasticity, balance disorders, and decreased endurance. The variety of levels of dysfunction could yield a variety of data on the level of physical activity among stroke patients.

The study conducted by Goncalves et al. (1) has its merits and can be of great help in shedding light on cardiovascular and musculoskeletal conditions that exist among stroke patients. A more rigorous study with homogeneity of participants, and exercise protocols based on clinical practice guidelines, would help in the generalizability of data, and help identify the benefits provided by increasing, and maintaining, increased physical activity levels among patients who have suffered a stroke.
