# Supplementary material for: COMMENTARY ON: COMPARISON OF MOTION SENSOR AND HEART RATE MONITOR FOR ASSESSMENT OF PHYSICAL ACTIVITY INTENSITY IN STROKE OUTPATIENT REHABILITATION SESSIONS: AN OBSERVATIONAL STUDY
Source: J Rehabil Med. 2024 Oct 9;56:41967. doi: 10.2340/jrm.v56.41967 (PMC11481306; doi:10.2340/jrm.v56.41967)
Supplement: Related texts

# Reply: RESPONSE TO THE COMMENTARY ON: COMPARISON OF MOTION SENSOR AND HEART RATE MONITOR FOR ASSESSMENT OF PHYSICAL ACTIVITY INTENSITY IN STROKE OUTPATIENT REHABILITATION SESSIONS: AN OBSERVATIONAL STUDY

**Authors:** Stéphanie GONCALVES, Stéphane MANDIGOUT, Morgane LE BOURVELLEC, Noémie C. DUCLOS

**Affiliations:** 1 University of Limoges, HAVAE, UR 20217, Limoges; 2 MOVE UR 20296, Poitiers University; 3 University of Bordeaux, INSERM, BPH, U1219, Bordeaux, France

We thank Felicianus Anthony Pereira et al. for their thoughtful commentary on our article. We appreciate the opportunity to clarify some key points that may have led to misunderstanding regarding the study’s primary objectives and design.

First, we wish to emphasize that the primary aim of our research was to compare the estimations of physical activity intensity between a motion sensor and a heart rate monitor in stroke patients during routine outpatient rehabilitation sessions. Our study was conducted in real-world clinical settings without implementing a specific exercise protocol or selecting patients based on clinical characteristics. This approach was intentional and designed to reflect actual practices in stroke rehabilitation in France rather than a controlled, standardized clinical trial environment.

Second, regarding the heterogeneity of participants, we acknowledge that stroke severity and related cardiac complications are essential factors in rehabilitation outcomes. However, our objective was to evaluate the tools used to estimate physical activity intensity under routine conditions, as typically encountered in outpatient physiotherapy settings. The diversity of the patient population in our study mirrors the typical range of chronic-phase stroke patients seen in everyday practice, offering valuable insights into the use of these tools in real-world rehabilitation scenarios.

Additionally, in the French healthcare system, the frequency and content of rehabilitation sessions are determined collaboratively between the patient and the physiotherapist. This reflects the typical practice of outpatient stroke rehabilitation in France.

We recognize the importance of standardized exercise protocols and homogeneity in patient selection for certain types of studies, particularly when exploring the clinical outcomes of rehabilitation interventions. However, this was not the focus of our research. For more details on the clinical characteristics of the stroke patients in our study and the specific exercises used during the rehabilitation sessions, we refer readers to a separate article we published recently, which specifically addresses these aspects in more depth (1).

In conclusion, our study contributes meaningful insights into the use of monitoring tools in stroke rehabilitation under routine clinical conditions. We appreciate the comments from Pereira et al. and hope this response clarifies the objectives of our study and its relevance to real-world clinical practice.

## References

[1] Goncalves S, Le Bourvellec M, Duclos NC, Mandigout S. Recommended moderate to vigorous physical activity levels for people in the chronic phase of stroke can be achieved in outpatient physiotherapy: a multicentre observational study. Top Stroke Rehabil 2024; Aug 22 [Online ahead of print]. 10.1080/10749357.2024.239244739172127
